# Preparation and Characterization of Self-Colored Waterborne Polyurethane and Its Application in Eco-Friendly Manufacturing of Microfiber Synthetic Leather Base

**DOI:** 10.3390/polym10030289

**Published:** 2018-03-08

**Authors:** Yulu Wang, Liqiang Jin

**Affiliations:** School of Leather Chemistry and Engineering, Qilu University of Technology (Shandong Academy of Sciences), Jinan 250353, China; jin-liqiang@163.com

**Keywords:** waterborne polyurethane, self-colored polyurethane, microfiber synthetic leather, eco-friendly manufacture, color fastness to washing

## Abstract

A novel self-colored waterborne polyurethane (SCPU) was synthesized and used in the preparation of a microfiber synthetic leather (MS-Leather) base in order to reduce the pollution caused by *N*,*N*-dimethylformamide (DMF) and dyes. The SCPU was prepared using the reaction of a reactive brilliant red K-2G with a waterborne polyurethane which was then extended via a first generation of hyperbranched poly(amine-ester). With the introduction of the dye, new absorption peaks at 1118 cm^−1^ [S=O], 1413 cm^−1^ [N=N], and 1635 cm^−1^ [C=N] appeared in the Fourier transform infrared (FTIR) spectrum of SCPU, and SCPU mean particle size increased to 162 nm. The X-ray diffraction (XRD) peak intensity of SCPU at 19.27° was 1310 cts. The thermal stability of SCPU at 200–280 °C was inferior to that of the control sample; however, it improved at temperatures above 360 °C. Finally, a free-dyeing MS-Leather base prepared by using SCPU without DMF was manufactured. It was found that the dyes were distributed mainly in the polyurethane matrix rather than in the microfibers. Moreover, the color changes of the base before and after being washed in both a water and a soap solution were 0.93 and 4.21, respectively. This indicated that the base’s washing color fastness to water was better than to a soap solution.

## 1. Introduction

Recently, the design and manufacture of leather substitutes or artificial leather have increased in Southeast Asia in order to meet the escalating demand for the insufficient output of genuine leather as well as ethical concerns over animal rights [1,2,3]. Among various leather substitutes, microfiber synthetic leather (MS-Leather) is becoming increasingly popular on account of its highly realistic leather-like appearance and touch. Additionally, its mechanical strength, chemical and thermal stability, quality homogeneity, and material utilization are superior to genuine leather [4,5]. Microfiber synthetic leather has found wide applications in the manufacture of shoes, garments, bags, and upholsteries in the aviation, automotive, and other industries [3,6].

Microfiber synthetic leather comprises a three-dimensional, reticulated, nonwoven fabric skeleton and a porous, polyurethane matrix [3,6]. The former is composed of numerous polyamide fibers which simulate the structure of natural collagen fibers. The latter supplies a feel and elasticity similar to genuine leather. In traditional manufacturing processes, the polyamide fibers are first dipped into a polyurethane solution containing *N*,*N*-dimethylformamide (DMF). The impregnated fabric is then transferred into water for the coagulation of polyurethane, in which the DMF dissolves into water and the polyurethane gathers together to form a continuous microporous film. In these processes, the use of DMF ensures the formation of a porous structure and the feel of the final product. However, workers in the industry face health risks as DMF can easily be absorbed through the skin or via the lungs and gastrointestinal tract [7]. Individuals who experience acute accidental overexposure to DMF can suffer from liver and kidney damage, while workers with long-term exposure to this chemical may face chronic gastrointestinal disturbances and liver damage [8,9]. Furthermore, DMF has been classified as toxic and potentially damaging to human fertility. In fact, DMF has been identified “as a substance of very high concern because of its CMR properties (CMR means carcinogenic, mutagenic, or toxic for reproduction)” [10,11].

As a universal solvent, about 50% of all DMF is used in synthetic leather factories in mainland China [7,12]. Unfortunately, significant levels of DMF have been discharged into the surrounding atmosphere and surface water [13,14]. DMF concentrations in some areas close to synthetic leather factories in industrial zones have been found to be higher than those of ordinary workplaces [7,13]. Therefore, not only the operators of synthetic leather factories but also the residents living around these factories face potential health threats related to DMF [7,13,15]. In order to avoid the damage of DMF, one of the most feasible solutions is the utilization of waterborne polyurethane. In fact, considerable attention has been paid to the utilization of waterborne polyurethane as a coating or finishing agent of MS-Leather and genuine leather [16]. However, few studies have been conducted on the use of waterborne polyurethane as the impregnated resin for the preparation of microfiber synthetic leather bases.

Besides the problems caused by DMF, the pollution of residual dye in the dyeing effluent of MS-Leather bases is also intractable. Generally, the color of MS-Leather articles is usually the most evident property first assessed by the customer, and thus good coloring is necessary for MS-Leather bases. However, the different chemical properties of polyamide microfibers and ordinary polyurethane matrices make the dyeing of MS-Leather bases difficult [3,17]. For the dyeing of polyamide microfibers, acid dyes are the most appropriate because stable ionic bonds can form between the protonated amino terminals of the polyamide molecules and the anions of the acid dyes [18,19,20]. Consequently, acid dyes are widely used in the coloration of MS-Leather bases. However, acid dyes are not suitable for the dyeing of polyurethane, another important part of a MS-Leather base. The soft segments in current polyurethane, which is used as a filling resin, consist of a high-molecular weight polyether or polyester, while the hard segments are diisocyanates linked through ethylene glycol or butanediol. Thus, there are no sufficient sites on polyurethane to bind strongly to acid dyes [3]. Additionally, the crystalline regions in either the soft segment or the hard segment of the filling resin can prevent the diffusion of dyes into the polyurethane matrix [21,22]. Therefore, the uptake of acid dye in polyurethane as well as the color fastness and uniformity of MS-Leather bases are relatively poor. Additionally, large amounts of dyes remain in the effluent and, consequently, are discharged to the surrounding environment [23]. To resolve these difficulties, the most common method is the use of more than one kind of dye for MS-Leather bases, which complicates the dyeing process and compromises the color levels in the final product [24,25]. Recently, studies have paid attention to the development of new additives or chain extenders with trialkylamine groups which aid in the adsorption and fixation of acid dyes [3,26,27,28,29,30,31]. In spite of this, the utilization rate of dye in the coloring of MS-Leather bases is not efficient or satisfactory.

In the present article, a synthesis of a novel self-colored waterborne polyurethane was attempted to resolve both the DMF and dye pollution with one stroke. If a waterborne polyurethane covalently bonded with dye molecules is used as a filling resin for the manufacture of MS-Leather bases, the use of DMF will be avoided. Additionally, MS-Leather bases will not require further dyeing, significantly decreasing the environmental pollution caused by dye discharge. Based on this concept, a given amount of hyperbranched poly(amine-ester) with terminal hydroxyl groups was used as a chain extender to prepare waterborne polyurethane with hydroxyl groups (WPU-H). This reacted with a reactive dye to obtain a self-colored waterborne polyurethane as the filler of MS-Leather bases. The synthesis of this novel self-colored waterborne polyurethane (SCPU) and its application in the preparation of MS-Leather bases are described below.

## 2. Experimental

### 2.1. Materials

Polyoxytetramethylene glycol with number molecular weight of 1000 (PTMG 1000) was purchased from Wanhua Chemical (Yantai, China). It was dehydrated for 24 h at 110 °C under vacuum prior to use. Isophorone diisocyanate (IPDI), 1,4-butanediol (BDO), 1-methyl-2-pyrrolidone (NMP), and acetone of analytical pure grade were purchased from Sigma Alderich (Shanghai, China) and used after dehydration with 4 Å molecular sieves for one week. Dimethylolpropionic acid (DMPA), triethylamine (TEA), sodium hydroxide, acetic acid, and dibutyltin dilaurate (DBTDL) were purchased from Sinopharm Chemical Reagent Co. Ltd. (Shanghai, China) and used as received. The dye reactive brilliant red K-2G (λ_max_ = 510 nm, CAS:12228-01-6) was supplied by Zhongcheng Chemical Co. Ltd. (Taizhou, China). Microfiber, nonwoven fabrics were provided by Zhejiang Hexin Holdings Ltd. (Jiaxing, China).

The first-generation hyperbranched poly(amine-ester) (HPAE-1) with hydroxyl value of 563 mg KOH/g was synthesized in our lab according to the method of reference [32,33], and all materials used were analytical-grade and purchased from Aladdin Industrial Corporation (Shanghai, China).

### 2.2. Synthesis of Self-Colored Waterborne Polyurethane (SCPU)

#### 2.2.1. Synthesis of Waterborne Polyurethane (WPU)

Fifty grams of PTMG was introduced into a flask equipped with a mechanical stirrer, nitrogen inlet, thermometer, and condenser with a drying tube. Then, 22.2 g of IPDI was added dropwise into the reactor at 80 °C, followed by 0.05 wt % of DBTDL as a catalyst. Two hours later, 4 wt % of DMPA dissolved in 1.5 times NMP was added into the system and reacted for 1.5–2 h. Subsequently, the temperature was decreased to 60 °C, and 1.91 g of BDO was introduced into the mixture and the reaction was continued for additional 30 min. After that, the mixture was neutralized with 2.32 g of TEA, and acetone was used to reduce the viscosity of the mixture. A given amount of deionized water was added to the flask to emulsify the product, and the acetone was removed under reduced pressure. The resulting product WPU was an emulsion with blue colour, and the polymer content was 30%.

#### 2.2.2. Preparation of Waterborne Polyurethane Modified by HPAE-1 (WPU-H)

The synthesis of waterborne polyurethane modified by HPAE-1 (WPU-H) was similar to the preparation of WPU, except that the chain extenders were 0.95 g BDO and 3.66 g HPAE-1.

#### 2.2.3. Preparation of Self-Colored Waterborne Polyurethane (SCPU)

**Figure 1:**
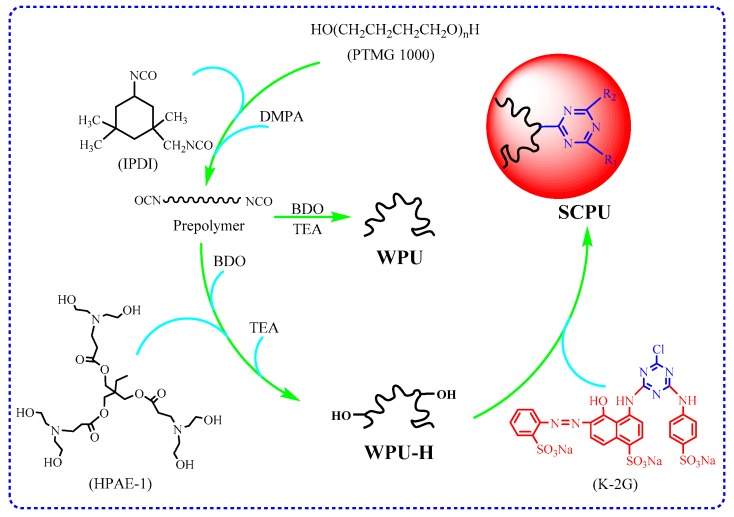
Synthesis procedures of waterborne Polyurethane (WPU), waterborne polyurethane modified by HPAE-1 (WPU-H), and self-colored waterborne polyurethane (SCPU). (Where IPDI: isophorone diisocyanate; DMPA: dimethylolpropionic acid; BDO: 1,4-butanediol; TEA: triethylamine; HPAE-1: the first-generation hyperbranched poly(amine-ester)).

A given amount of WPU-H was added into a flask with a stirrer, a thermometer, and a reflux condenser; its pH was adjusted to 9 by using 10 wt % of sodium hydroxide solution. Three percent by weight of reactive brilliant red K-2G (with respect to polymer of WPU-H) was dissolved in deionized water and introduced into the mixture at 60 °C for 3 h. Finally, self-colored waterborne polyurethane (SCPU) containing 20 wt % of colored polymer was obtained. The preparation procedures of waterborne polyurethane synthesized above are illustrated in Figure 1.

### 2.3. Characterizations of Polyurethanes

#### 2.3.1. FTIR Spectrum

WPU, WPU-H, and SCPU were dissolved into acetone, and the concentrations of polymers were all 1 wt %. A little amount of the sample above was dropped onto a piece of preformed KBr pellet and dried completely under an infrared light. Infrared spectra of WPU, WPU-H, and SCPU films were obtained by TENSOR-27 (Bruker, Ettlingen, Germany) over a range of 1000–4000 cm^−1^ with nominal resolution of 2 cm^−1^ at room temperature.

#### 2.3.2. Particle Size Analysis

Samples of WPU, WPU-H, and SCPU were diluted by deionized water to a given concentration, and their particle sizes were measured by Zetasizer Nano ZS90 (Malvern, Malvin City, UK) at room temperature.

#### 2.3.3. X-ray Diffraction Characterization

The polyurethane solutions of WPU, WPU-H, and SCPU were cast on plastic culture dishes and dried at room temperature for 72 h. Then, they were heated at 80 °C under vacuum conditions to a constant mass. Finally, the resulting films were peeled from the plates and stored in vacuum desiccators before X-ray diffraction (XRD) characterization and thermogravimetric (TG) analysis.

XRD patterns were characterized by using a PANalytical X’Pert System (Philips, Almelo, The Netherlands). The sample was placed randomly on a flat brass sample holder with an O-ring sealed cover which provided an airtight atmosphere. Cu K_α1_ radiation (λ = 1.542 Å) was generated at a voltage of 40 V and electric current of 30 mA. The XRD data were recorded in the Bragg’s angle 2θ range of 5–50° with a scanning step size of 0.02° and a time of 1 s per step. 

#### 2.3.4. Thermal Performances

The thermogravimetric (TG) characteristics of polyurethane films prepared above were investigated by a Q500 thermogravimetric analyzer (TA Instruments, Milford, MA, USA). The samples were put in ceramic pans and heated from room temperature to 600 °C at a heating rate of 10 °C/min in a nitrogen atmosphere.

### 2.4. Preparation and Characterization of MS-Leather Base

A piece of nonwoven, microfiber fabric with an area of about 5 cm × 10 cm was peeled 20 times in a sodium hydroxide solution (10 wt %) at 90 °C for 60 min. After being washed adequately in deionized water, the split, microfiber fabric was dried at 80 °C for 3 h. Then, the dried, split microfiber fabric was dipped in SCPU and flattened by a SS-Spreader onto a glass plate in order to squeeze out the superfluous resin. Then, the impregnated microfiber fabric was quickly put into a 0.20 mol/L acetic acid solution for the solidification of waterborne SCPU. Five minutes later, the fabric with the colored resin was taken out and dried at 80 °C for 3 h. As a result, a colored MS-leather base was achieved, which was marked as CLB. The content of dye in CLB was calculated by Equation (1):

(1) $$C_{CLB}=\left(W_{2}-W_{1}\right)\times 3\%/W_{2}$$

where *C_CLB_* is the content of K-2G in CL, in mg/g; *W*_2_ and *W*_1_ are the weight of CLB and the dried, split microfiber fabric. respectively, in g.

Another base, denoted as DLB, was prepared using WPU-H in a similar procedure. It was dyed in a 20-time dye solution at 90 °C for 3 h per the traditional dyeing process, and the dosage of K-2G was 4 wt % of DLB. The pH of the solution was adjusted to about 4 at the end of dyeing for a better fixation. The absorbance of the solution during dyeing was measured by a Shimadzu spectrophotometer UV3600 (Shimadzu, Kyoto, Japan) in order to calculate the uptake rate of the dye. The dye content in DLB was calculated using Equation (2):

(2) $$C_{DLB}=\frac{m\times 4\%\times R_{U}\%}{m+m\times 4\%\times R_{U}\%}$$

where *C_DLB_* is the content of K-2G in DLB in mg/g; *m* is the weight of DLB in g; *R_U_* is the % dye uptake at equilibrium.

The surface and cross-sectional morphologies of CLB and DLB were observed by a Digital Microscope VHX-2000 Series (Keyence, Osaka, Japan).

The L*, a*, b*, C*, and DE* coordinates of CLB and DLB were measured using a X-rite 8200 spectrophotometer, and the viewing conditions used were illuminant D65, 10° standard observer. Three points on CLB or DLB were selected randomly for measurement, and the average value was evaluated.

Additionally, the CLB was washed with deionized water or a 2 g/L soap solution, according to international standard ISO 11642 in order to evaluate its color fastness. The color coordinates before and after washing were measured using a X-rite 8200 spectrophotometer.

## 3. Results and Discussion

### 3.1. FTIR Analysis

**Figure 2:**
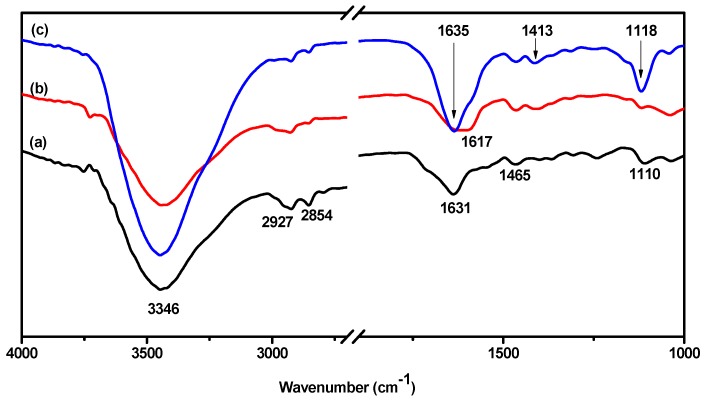
FTIR spectrum of waterborne polyurethane: (**a**) WPU; (**b**) WPU-H; and (**c**) SCPU.

As shown in Figure 2, the chain structures of WPU, WPU-H, and SCPU were confirmed by the FTIR spectrophotometer. The FTIR spectrum of WPU showed the typical absorption peaks of polyurethane at 3346 cm^−1^ [*ν*(N-H)], 2854 cm^−1^ [*ν*(C-H)], 1631 cm^−1^ [*ν*(C=O)], 1465 cm^−1^ [δ(N-H)], and 1110 cm^−1^ [*ν*(C-O-C)] [34,35]. The NCO absorption peak at about 2270 cm^−1^ disappeared in the spectrum, showing that NCO groups had completely reacted during the reaction.

The FTIR spectrum of WPU-H was very similar to that of WPU, despite a shift of absorption peaks from 1631 cm^−1^ [*ν*(C=O)] to 1617 cm^−1^. This was probably because the unreacted hydroxyl groups in HPAE-1 strengthened the hydrogen bond interaction of hard segments of polyurethane [36] and moved the absorption peak of C=O from a high wavenumber to a low wavenumber. The typical absorption band of OH at 3400 cm^−1^ was not obvious and might have overlapped with the absorption band of N-H [37]. It was manifested that waterborne polyurethane containing free hydroxyl groups (WPU-H) was synthesized.

The FTIR absorption bands of SCPU were different from those of WPU and WPU-H. Compared with WPU-H, a distinct absorption peak at 1118 cm^−1^ appeared, which was attributed to the asymmetric stretching vibration of S=O in sulfonic acid groups of dye molecules [38]. The absorption at 1413 cm^−1^ was the stretching vibration peak of N=N. According to the shape and intensity, the absorption peak at 1635 cm^−1^ was different from the peak of WPU at 1631 cm^−1^. It was attributed to the absorption of C=N of the dye. Moreover, the intensity of the absorption peak of N-H at 3346 cm^−1^ was strengthened by the introduction of the dye. On the basis of the results above, the self-colored waterborne polyurethane was successfully prepared.

### 3.2. Particle Size Distribution

**Figure 3:**
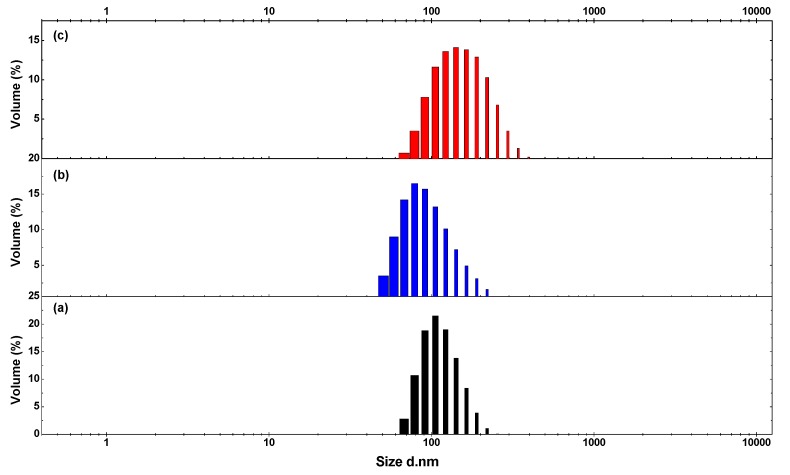
Particle size distribution of waterborne polyurethane: (**a**) WPU; (**b**) WPU-H; and (**c**) SCPU.

The particle sizes and their distribution of WPU, WPU-H, and SCPU are shown in Figure 3. The particle sizes of WPU were between 68 and 255 nm, with an average of 116 nm, proving that the WPU prepared above was a nanoscale emulsion. Compared with WPU, the particle size distribution of waterborne polyurethane modified with HPAE-1 (WPU-H) increased. The range of WPU-H particle sizes was 43–295 nm, and its average particle size decreased to 100 nm as a result of the modification of HPAE-1. A possible explanation is that the introduction of ramified HPAE-1 increased the intermolecular interaction of the polymers, and thus larger particles formed [39]. Hence, the particle size distribution of WPU-H became broader. However, the average particle size of waterborne polyurethane is governed mainly by its hydrophilicity [40,41]. Even though the content of carboxylic groups in WPU and WPU-H was the same, the redundant hydroxyl groups of WPU-H would increase its hydrophilicity. Therefore, the mean particle size of WPU-H was smaller than that of WPU.

Compared with WPU-H, the particle size range and average size value of SCPU increased to 68–396 nm and 162 nm, respectively. This was because the hydroxyl group of WPU-H reacted with the active chlorine in the reactive brilliant red K-2G, and the dye molecule was grafted onto the polyurethane. However, a large steric hindrance of dye molecule decreased the flexibility of the polymer, and the dispersion effect was reduced [42].

### 3.3. XRD Characterization

**Figure 4:**
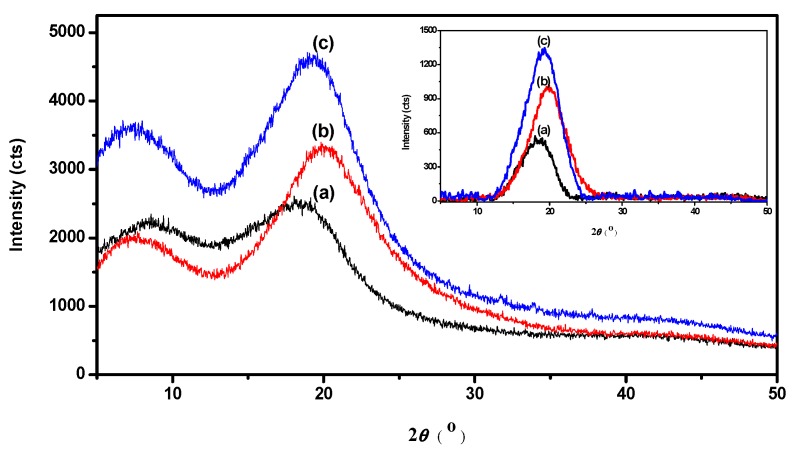
XRD patterns of (**a**) WPU; (**b**) WPU-H; and (**c**) SCPU. The inset shows the data by background correction.

The XRD patterns of WPU, WPU-H, and SCPU are shown in Figure 4 and display typical amorphous characteristics [3,43]. Compared with WPU, the 2θ degree of WPU-H shifted from 18.60° to 20.03°. This was because the short-range-order arrangement of the polymer chain orientated better after the modification of HAPE-1. However, the 2θ degree of SCPU decreased slightly to 19.27°, which was attributed to the steric hindrance of the grafted dye molecules. Generally, the higher the intensity of the diffraction peaks, the higher the crystallinity in the polyurethane [44]. Furthermore, the crystallinity was affected greatly by the hydrogen bond interaction [45]. The XRD peak intensity of WPU was 595 cts, while that of WPU-H increased to 1029 cts. This was because the hyperbranched poly(amine-ester) was distributed mainly in the hard segments of the polyurethane, and the hydrogen bonds interacted among the hydroxyl groups on the HPAE-1 and urethane groups. Therefore, the micro-phase separation of WPU-H, compared to WPU, was strengthened. After reacting with the dye molecules, the micro-phase separation of the hard and soft segments of SCPU was further increased as a consequence of the higher steric hindrance resulting from the bulkiness of dye molecules. Thus, the XRD peak intensity of SCPU increased to 1310 cts. The XRD patterns also confirmed that the dye molecules were grafted successfully onto the polyurethane, which agreed with the results of FTIR and particle size analysis.

### 3.4. Thermal Properties

**Figure 5:**
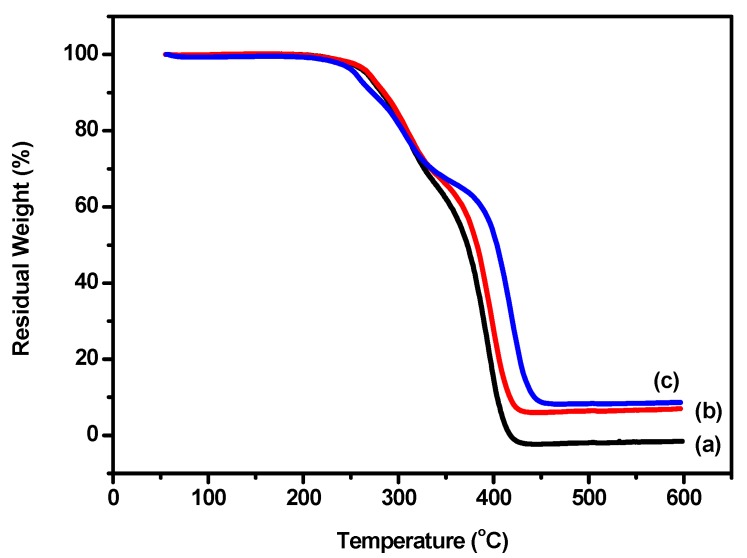
Thermogravimetric (TG) curves of waterborne polyurethane: (**a**) WPU; (**b**) WPU-H, and (**c**) SCPU.

A thermogravimetric (TG) analyzer was applied to evaluate the thermal stability of all types of polyurethane prepared above. TG curves of cured films of WPU, WPU-H, and SCPU are shown in Figure 5. The 5% weight loss for WPU was at 266 °C. The 50% and 80% weight losses were at 372 °C and 396 °C, respectively, while the WPU decomposed completely at 418 °C. In the case of WPU-H, 5%, 50%, and 80% weight losses occurred at 270 °C, 383 °C, and 406 °C, respectively. This indicated that the thermal stability of polyurethane increased after being modified by HPAE-1. Generally, the thermal decomposition of polyurethane presented two stages. The first step was the degradation of the hard segment of polyurethane, whilst the second step was the decomposition of its soft segment [35,46]. Thus, the thermal stability of the hard segment of WPU-H was higher than that of WPU because of the strengthening of hydrogen bond interactions via the free hydroxyl groups on HPAE-1. The results of TG corresponded to the records of particle size distribution and FTIR spectra analysis above.

The thermal stability of SCPU at 200–280 °C was inferior to that of WPU-H according to the TG curves shown in Figure 5b,c. This was a result of the grafting of dye molecules on polyurethane; their bulkiness caused higher steric hindrance and consequently decreased the hydrogen bond interaction of the hard segments. However, the thermal stability of SCPU was higher than that of WPU-H when the temperature was above 360 °C, because of the influence of some unreacted dye molecules distributed and hydrogen bonded in the hard segment of the polyurethane [35,46].

### 3.5. The Micromorphologies of MS-Leather Base

**Figure 6:**
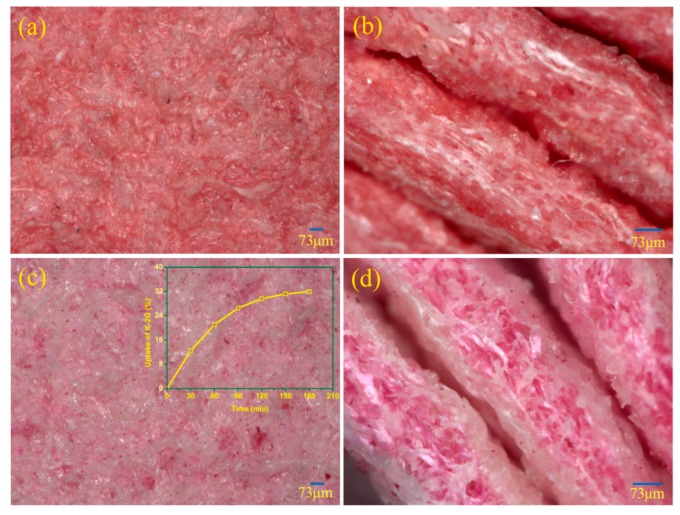
The micromorphologies of the MS-Leather bases: (**a**) surface of CLB; (**b**) cross section of CLB; (**c**) surface of DLB; and (**d**) cross section of DLB. The inset was the uptake curve of K-2G in the coloration of DLB.

Generally, waterborne polyurethane contained little or no solvent and was unable to coagulate like a solvent base polyurethane in the manufacture of a MS-Leather base. Therefore, an acetic acid solution was selected as the solidification agent of waterborne polyurethane to prepare MS-Leather bases. The surface and cross-sectional micro-morphologies of CLB and DLB observed by Digital Microscope VHX-2000 are shown in Figure 6. As shown, the polyurethane coagulated well on the surfaces of bases and distributed evenly around the microfibers. This was because the waterborne polyurethane emulsion was composed of ionomer dispersions, whose stability was greatly influenced by the pH, as well as the selection and dosage of the neutralizing agent [41]. In an acetic acid solution, the neutralizing agent (triethylamine) of waterborne polyurethane reacted preferentially with acetic acid, and the polyurethane particles became unstable as a result of losing counter-ions. Moreover, the pH of the acetic acid solution was very low, which made the polyurethane precipitate quickly from the water. Compared with traditional manufacture processes of MS-Leather bases, no solvent such as DMF was used in our experiments, and consequently no harm caused by DMF was introduced. The waterborne polyurethane featured in this paper as an organic, solvent DMF-free manufacturing process of MS-Leather was shown to be more workable and eco-friendly.

Moreover, the surface and cross section of CLB without dyeing appeared red because of the color of SCPU. It can be easily observed from Figure 6b that the dye distributed evenly in the polyurethane matrix rather than in the microfibers. In contrast, the dyes were mainly adsorbed in the microfibers of DLB which was dyed using a traditional dyeing method, as shown in Figure 6d. As a result, the surface color of DLB was paler or lighter than that of CLB. The inset in Figure 6c was the uptake curve of K-2G in the coloration of DLB, and the equilibrium dyeing rate was only 34.32%. Similarly, in the practical coloration of an MS-Leather base, a large amount of superfluous dyes remained in the dyeing solution and was discharged as effluent.

The MS-Leather base prepared by using SCPU would not be dyed further as a traditional base. The dye contents of CLB and DLB are listed in Table 1 and were 7.80 mg/g and 13.54 mg/g, respectively. Their color coordinates of C* and DE* are also listed in Table 1; the former reflects the color saturation of the samples and the latter the color difference of the samples. Interestingly, the average values of C* and DE* of CLB were much higher than those of DLB, which indicated that the color of CLB was much brighter than that of DLB. In other words, the utilization rate of dye to prepare a MS-Leather base by using self-colored waterborne polyurethane was much more efficient than that of traditional dyeing methods. Furthermore, the manufacturing process of the MS-Leather base by using SCPU hardly discharged effluent containing dye. Therefore, the less-dye process featured in this paper provides an environmentally friendly option for the manufacture of MS-Leather bases.

### 3.6. The Color Fastness to Washing of CLB

MS-Leather bases prepared by SCPU were washed with either deionized water or a soap solution, after which the color coordinates were measured in order to survey their washing fastness. The color coordinates are shown in Table 2, and the ΔE is the difference of DE average value before and after being washed. As shown in Table 2, the a* values of CLB were positive and commensurate to each other, which indicated the CLB was a uniform red in color. After being washed in water, the a* values of CLB decreased slightly, in comparison to a significant decrease after being washed in the soap solution. The corresponding difference ΔE was 0.93 and 4.21 color difference units, respectively. In fact, there is a relationship between the color change and the grey scale rating, which is the criterion of color fastness based on ISO 105-A05 [47]. The color change (ΔE) of a 4–5 grey scale rating is 0.8 ± 0.2 color difference unit, while the ΔE of a 4 grade rating is 1.7 ± 0.3 color difference units. Thus, the color fastness of CLB against water was classified as a 4–5 grade, which corresponds to limited human visual discrimination. However, the color variation of CLB washed by the soap solution was 4.21, which was less than 4.8 ± 0.5 (2–3 grade) and classified as a 3 grade. This demonstrated that the washing color fastness of CBL against water was better than against the soap solution. Nevertheless, some dye molecules dissolved into water when CLB was washed with a soap solution, and consequently the color of the base faded. Some dye molecules had not been grafted onto the polyurethane polymer; thus, they dispersed in the polyurethane matrix. After being solidified and dried, the polyurethane matrix on CLB had little hydrophilicity, preventing the dissolution of dyes even though the matrix contained some carboxyl groups. However, the hydrophilic ability of polyurethane with carboxylic groups would increase in an alkaline soap solution. Therefore, compared with washing in water, the uncombined dyes were more easily washed out of the base in the soap solution.

## 4. Conclusions

The synthesis, characterization, and application of a novel self-colored waterborne polyurethane was reported in the present study. The dye molecules were successfully grafted onto the waterborne polyurethane chain, which was demonstrated by new infrared absorption peaks at 1118 cm^−1^, 1413 cm^−1^, and 1635 cm^−1^ on the FTIR spectrum of SCPU. Following the introduction of dye, the hydrophilicity of polyurethane decreased slightly, broadening the particle size distribution and increasing the mean particle size. Furthermore, the micro-phase separation of polyurethane was increased via the steric hindrance and hydrogen-bonding action of the dye molecules, which was confirmed by the increased intensity of the XRD diffraction peak. On the basis of the analysis of TG, it was proven that some dye molecules were not grafted onto the polymer chain while hydrogen-bonded in hard segments of polyurethane. However, the unreacted dyes increased the thermal stability of polyurethane.

An eco-friendly process was successfully designed to prepare a MS-Leather base by using SCPU, in which no DMF was used and no further dyeing was required. In this process, the waterborne polyurethane coagulated well in a 0.2 mol/L acetic acid solution. The color of the base prepared with SCPU was brighter with a reduced dye dosage, as the dye molecules were distributed mainly in the polyurethane matrix rather than in the microfibers. Importantly, the discharged effluent of this process contained hardly any dye. The color variation of the base before and after being washing with water was 0.93, which was classified as a 4–5 grey scale rating. This indicates that the base prepared by SCPU had good color fastness to washing with water. However, the washing color fastness of the base to a soap solution needs to be improved.

## Figures and Tables

**Table 1:** The dye contents of CLB and DLB and their color coordinates.

Sample	Content of Dye (mg/g)	C*	$\bar{C}$ ^a^	DE*	$\bar{DE}$ ^b^
CLB	7.80	53.88	53.75	82.33	82.26
		53.81		82.32	
		53.57		82.13	
DLB	13.54	39.04	39.43	61.72	62.28
		39.26		62.03	
		39.99		63.08	

^a^ Average value of C*; ^b^ average value of DE*.

**Table 2:** The color coordinates of CLB before and after being washed.

Sample	L*	a*	b*	DE*	$\bar{DE}$ ^a^	ΔE
CLB	56.78	50.58	17.95	82.33	82.26	/
	56.94	50.76	17.87	82.32		
	56.69	50.52	17.80	82.13		
CLB washed by water	56.82	50.29	17.51	81.73	81.33	0.93
	56.71	49.73	18.02	81.21		
	57.02	49.78	17.72	81.06		
CLB washed by soap solution	56.99	47.99	14.70	78.29	78.05	4.21
	57.41	47.79	14.56	77.82		
	56.97	47.78	14.72	78.05		

^a^ Average value of DE*.
